# Synthesis and Characterization of Photoresponsive Macromolecule for Biomedical Application

**DOI:** 10.3389/fchem.2018.00217

**Published:** 2018-07-02

**Authors:** Juan Pang, Ziyu Gao, Long Zhang, Huiming Wang, Xiaohong Hu

**Affiliations:** School of Material Engineering, Jinling Institute of Technology, Nanjing, China

**Keywords:** azobenzene, photo switcher, photoresponsive macromolecule, biomedical application, hyaluronic acid

## Abstract

Azobenzene, a photo switcher, has attracted increasing interest due to its structural response to photo stimulus in the field of information science and chemical sensing in the recent decades. However, limited water solubility and cytotoxicity restrained their applications in the biomedical field. In research, HA-AZO has been designed as a water soluble photo switcher in biomedical application. Synthesized HA-AZO had good water-solubility and a stable π-π^*^ transition absorbance peak trans-isomer. With exposure to UV, transformation from trans-isomer to cis-isomer of HA-AZO could be realized according to UV spectra. Reversely, trans-isomer could be gradually recovered from cis-isomer in the dark. Simultaneously, quick response and slow recovery could be detected in the process of structural change. Moreover, repeated illumination was further used to detect the antifatigue property of HA-AZO, which showed no sign of fatigue during 20 circles. The influence of pH value on UV spectrum for HA-AZO was investigated in the work. Importantly, in acid solution, HA-AZO no longer showed any photoresponsive property. Additionally, the status of HA-AZO under the effect of UV light was investigated by DLS results and TEM image. Finally, *in vitro* cytotoxicity evaluations were performed to show the effects of photoresponsive macromolecule on cells.

## Introduction

Photoresponsive molecules, which cause reversible changes, i.e., their chemical and physical properties changes due to structural change with a light stimulus, have attracted increasing interest in the field of information science and chemical sensing in the recent decades (Sun et al., 2012; Li et al., 2014; Yuan et al., 2014; Kim et al., 2015). Among photo molecular switches, azobenzenes have been intensively investigated due to the precise spatiotemporal control (Li et al., 2014; Kim et al., 2015). Upon exposure to UV light, azobenzenes can isomerize from the trans-form to the cis-form (Henzl et al., 2006; Kumar and Suresh, 2009; Schmidt et al., 2010). However, the cis-form is unstable and liable to reversely isomerize to trans-form under the action of visible light or slight heat (Henzl et al., 2006; Kumar and Suresh, 2009; Schmidt et al., 2010). Given the fact that cis- and trans-isomers of azobenzenes have a different spatial arrangement of the aromatic moieties, physical and chemical properties of azobenzenes varied a lot, some examples being π-π stack interaction among molecules, dipole moments, and surface wettability (Li et al., 2014; Yuan et al., 2014; Lin et al., 2016, 2017). Consequently, azobenzenes became particularly popular as photoresponsive chromophores in a number of research fields including biomedical fields (Deka et al., 2015; Liu et al., 2015; Bian et al., 2016a,b). Recently, on account of photo-controlled reversible supermolecular interaction between azobenzenes and cyclodextrins, hydrogels as well as films with controllable pores or passages crosslinked by the supermolecular interaction have been designed and synthesized to respond to photo-stimuli from external environment (Chen et al., 2009; Chiang and Chu, 2015; Wang et al., 2015). In addition to these reports, visible light driven azobenzene-based photo-switching molecules have been theoretically designed by different substitute groups (Pang et al., 2014, 2016; Ye et al., 2015).

On account of aqueous physiological environments, chemicals in the application of biomedicine-related fields should have aqueous dissolvability (Gao et al., 2016; Wang et al., 2016; Gu et al., 2017; Ha et al., 2018). From this point, the application of azobenzene in the biomedicine-related field is restricted not only due to its low aqueous dissolvablity but also due to cytotoxicity in water. In order to solve this problem, a water-soluble photo switch was designed. From previous research, some natural polysaccharides such as hyaluronic acid (HA), chitosan, chondroitin sulfate (CS) and the like is proven to be water soluble and have good biocompatibility especially for HA, which plays an important role in the organization and stabilization of ECM, cell proliferation, and differentiation (Hu et al., 2011; Hu and Gong, 2016). Moreover, HA can lubricate joints, adjust permeability of vessel walls, improve transport of protein and ions, and accelerate wound healing (Yue et al., 2017; Andreasen et al., 2018). Hence, HA is assumed to be an ideal macromolecule or material in biomedical application. In view of these facts, a photoresponsive macromolecule was designed using HA as a backbone and AZO as a side chain in this work.

In consideration of reactivity and carboxyl groups on HA molecules, p-aminoazobenzene was grafted onto the main chain of HA to obtain a photoresponsive macromolecule. In order to clarify the properties of the photosensitive macromolecules, molecular responsive properties as a function of irradiation time, pH value, and recovered time as well as fatigue resistance of the responsive property was characterized in the research. As a macromolecule, its molecular aggregation in water is also important to evaluate the interaction between molecules and provide potential material for biomedical application. Therefore, the macromolecule with different responsive status in water was tracked by dynamic light scattering (DLS) and molecular aggregation in water was also confirmed by transmission electron microscope (TEM) images. Finally, cell viability and apoptosis profile was used to evaluate the cytotoxicity of photoresponsive macromolecule.

Although azobenzenes have been intensively investigated, work still needs to be done to obtain a biocompatible photoresponsive macromolecule for biomedical application, characterize their structure and assess their properties including cytotoxicity properties.

## Experimental section

### Materials

1-Ethyl-3-(3-dimethylaminopropyl) carbodiimide hydrochloride (EDC), N-hydroxysuccinimide (NHS), 2-morpholinoethane sulfonic acid (MES), and p-aminoazobenzene (AZO) was purchased from Aladdin. Hyaluronate acid (HA, M_w_ = 1,000 kDa) was obtained from Shandong Furuida Co., China. Trypsin, Dulbecco's modified Eagle's medium (DMEM), fluorescein diacetate (FDA), and 3-(4, 5-dimethyl) thiazol-2,5-dimethyl tetrazolium bromide (MTT) were obtained from Sigma. Fetal bovine serum (FBS) was purchased from Sijiqing biotech. Co., China. All other reagents and solvents were of analytical grade and used as received.

### Synthesis of HA-AZO

HA-AZO was synthesized by amidation between HA and aminoazobenzene (AZO). Briefly, 500 mg of EDC and 200 mg of NHS were successively added to 10 mL of 2% HA solution with magnetic stirring. Meanwhile, 10 mg of AZO was dissolved in 10 mL DMF. After 30 min, 100 mg of MES was further added to HA solution. HA solution and AZO solution were subsequently mixed together and the reaction was maintained for 5 h at room temperature. Then, the final solution was dialyzed with a dialysis bag of 10 kDa cut-off molecular weight for 3 days to remove unreacted chemicals and byproduct of small molecular weight. Finally, HA-AZO was obtained by freeze drying at −60°C at a pressure of 7–8 Pa.

### Characterization of HA-AZO

HA-AZO was characterized by _1_H nuclear magnetic resonance (_1_H NMR, Bruker, AV500) using D_2_O as solvent. HA-AZO water solution, AZO water solution, and AZO DMSO solution was characterized by UV spectroscopy (Cary 50). UV lamp (10 W) was used as photosource to induce tran-to-cis transition of AZO domain. In order to track the structural change of molecules, real-time UV spectra as a function of irradiation time and recovery time was recorded. Repeated irradiation and recovery method were applied to demonstrate the fatigue resistance of molecules. Macromolecule status in water solution was characterized by dynamic light scattering (DLS, nano ZS) and transmission electron microscope (TEM, Philips, Tecnai 12).

### Cytotoxicity evaluation of HA-AZO

HUVEC cells were incubated in a humidified atmosphere of 95% air and 5% CO_2_ at 37°C. The used cells were detached using 0.25% trypsin in PBS for the experiment. Simultaneously, HA-AZO and AZO were dissolved in DMEM with certain concentration separately with the same molar ratio. Then, 100 μL of above-mentioned solution was added into each well of 96-well culture plate, and into which the 100 μL cell suspension containing 5,000 cells was subsequently added. Cells were characterized as a function of cultural time. MTT assay was used to characterize cytoviability. Briefly, 20 μL MTT was supplemented into wells of the culture plate, which were then put back to continually culture cells for another 4 h. Two hundred microliters DMSO was added to dissolve the formed formazan pigment. The absorbance of above solution at 490 nm was recorded by a microplate reader (Infinite M200 PRO).

Besides MTT assay, apoptosis profile of cells was characterized by PI/FITC double staining (Annexin V). Briefly, 1 mL of above-mentioned HA-AZO solution or AZO solution was added into each well of 12-well culture plate, into which 1 mL cell suspension containing 200,000 cells was subsequently added. Cells of each well were detached using 0.25% trypsin without EDTA after culturing for 24 h separately. Detached cells were washed with 1 × binding buffer, resuspended in 500 μL 1 × binding buffer, and subsequently ordinal stained by PI solution and FITC solution for 15 min. The stained cells detected by flow cytometry (FCM, BD, C6) within 1 h in dark.

### Statistical analysis

Data were analyzed using the *t*-test for differences. Results were reported as means ± standard deviation. The significant level was set at *p* < 0.05.

## Result and discussion

### Synthesis of HA-AZO

**Figure 1:**
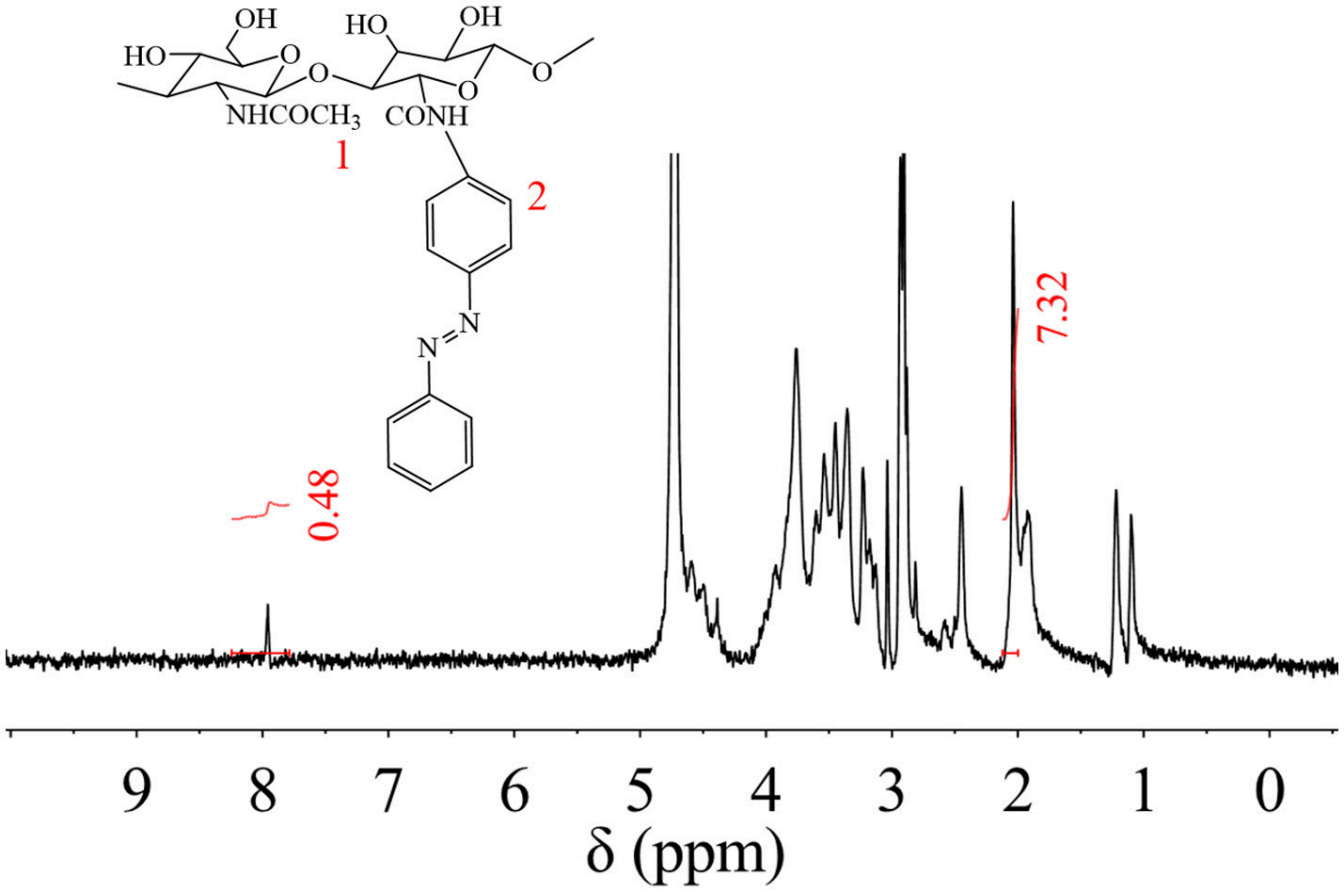
_1_H NMR spectrum of HA-AZO.

Synthesized HA-AZO was characterized by _1_H NMR as shown in Figure 1. The details of chemical shift are listed as follows: the chemical shift at 1.9 ppm is attributed to the protons of CH_3_-O at 1 position, the chemical shifts from 3.1 to 4.0 ppm are attributed to the protons of pyranose ring, and the chemical shift at 7.9 ppm is attributed to the protons of benzene ring of AZO at 2 position. The chemical shift at 2 position confirmed successful grafting of AZO onto HA main chain. Besides qualitative analysis, _1_H-NMR provided quantitative information since areas of resonance peaks are proportional to number of protons. According to areas in Figure 1 at 1 and 2 positions, the degree of substituent (DS) could be calculated using the average proton intensity ratio of the protons of benzene ring/CH_3_-O, which is 2.2% (per two pyranose ring).

### Characterization of HA-AZO

**Figure 2:**
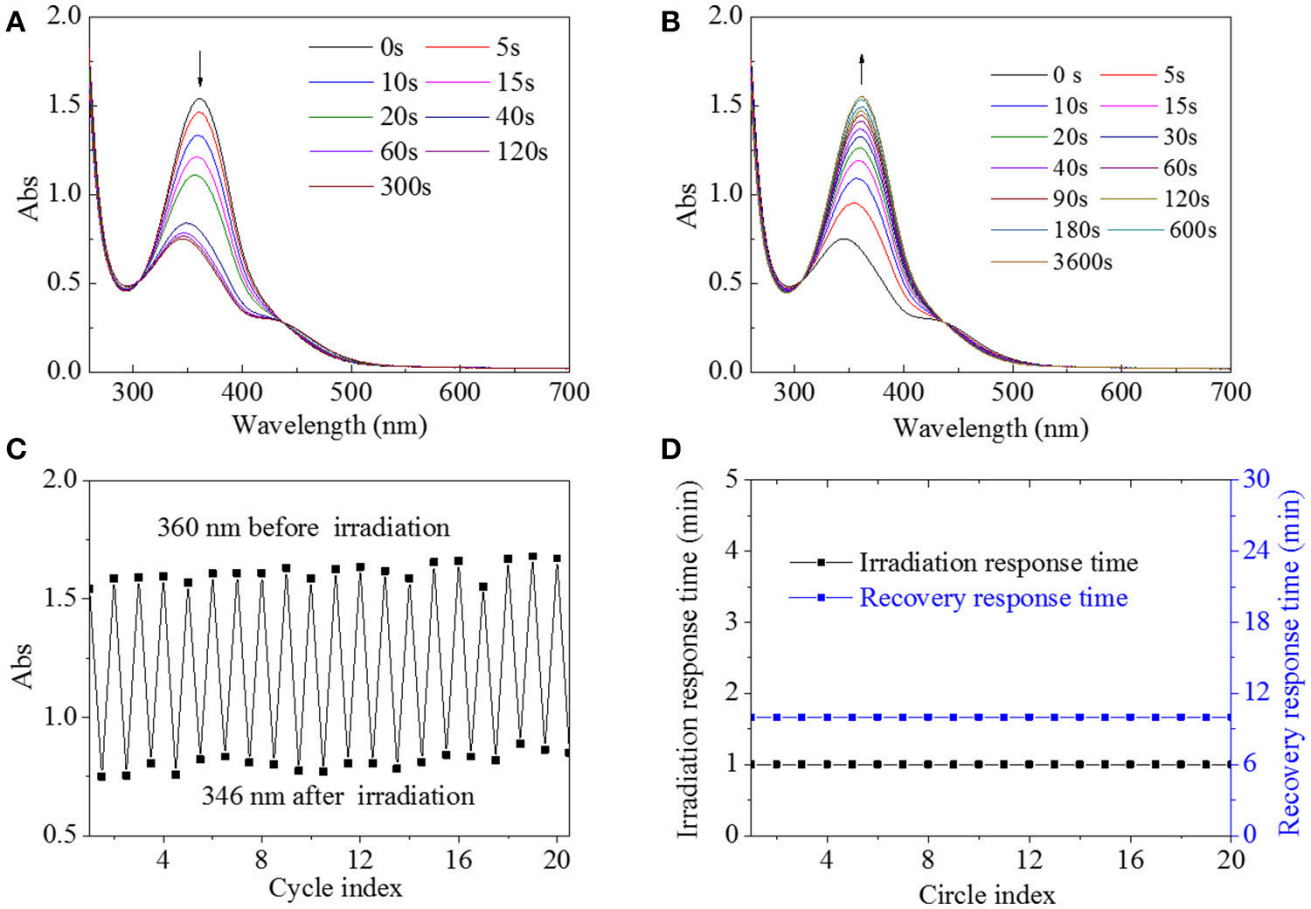
UV spectra of HA-AZO water solution as a function of irradiation time **(A)** and recovery time **(B)**. **(C)** Absorbance at 360/346 nm of HA-AZO water solution as a function of circle index. **(D)** Irradiation response time and recovery response time in dark as a function of circle index.

It was found that HA-AZO had good water-solubility. Hence, HA-AZO water solution was used for the following characterization. Firstly, UV spectra of HA-AZO water solution as a function of irradiation time and recovery time were shown in Figures 2A,B. In one aspect, the peak at 360 nm belonging to π-π^*^ transition for trans-isomer decreased significantly and the peak at 420 nm belonging to n-π^*^ transition for cis-isomer increased a little with irradiation time until 60 s, which was an obvious sign to indicate that the trans-form had been transferred to the cis-form (Figure 2A). In another aspect, without irradiation, the peaks at 360 and 420 nm were gradually recovered to their original state within 10 min, which indicated that the recovery of trans-form for AZO domain of HA-AZO was realized (Figure 2B). Structural change upon photo guaranteed the molecular responsive property for HA-AZO. Moreover, rapid transfer from trans to cis upon photo exhibited quick responsive time for the macromolecule, and simultaneously gradual recovering process indicated enough controllable time. Effective structural change, rapid responsive time, and enough operation time are all desirable properties for a macromolecule photo switch. As a contrast, UV spectra of AZO water solution showed no change after irradiation without any change of isomer (Figure s1), although AZO DMF solution showed some photo-responsive properties (Figure s2). Secondly, fatigue resistance of molecular switch was essential for its actual application, which was evaluated by repeated irradiation and recovery and characterized by UV spectrum as shown in Figures 2C,D. It was found that the minimum absorbance at 346 nm for HA water solution after irradiation was stabilized at 0.7–0.8 regardless of circle time, and simultaneously maximum absorbance at 360 nm either initial or after recovery was stabilized at 1.5–1.7 regardless of circle time (Figure 2C). The results showed that cis-form could be stable and exist after irradiation, whereas the trans-form could be recovered after removal of irradiation. Furthermore, the response time with irradiation was stable at 1 min, and the recovery time was stable at 10 min regardless of circle time (Figure 2D). From these results, it is inferred that HA-AZO has no sign of fatigue or phenomenon of photobleaching. As a contrast, even in DMF, the photoresponsive property weakened as a function of circle time (Figure s3A), and furthermore, the response time and recovery time shortened with increase of circle time (Figure s3B), which was unfavorable for its application as a photo switch. By contrast, HA-AZO had obvious superiority to AZO molecule as a photo switch, especially in water solution.

**Figure 3:**
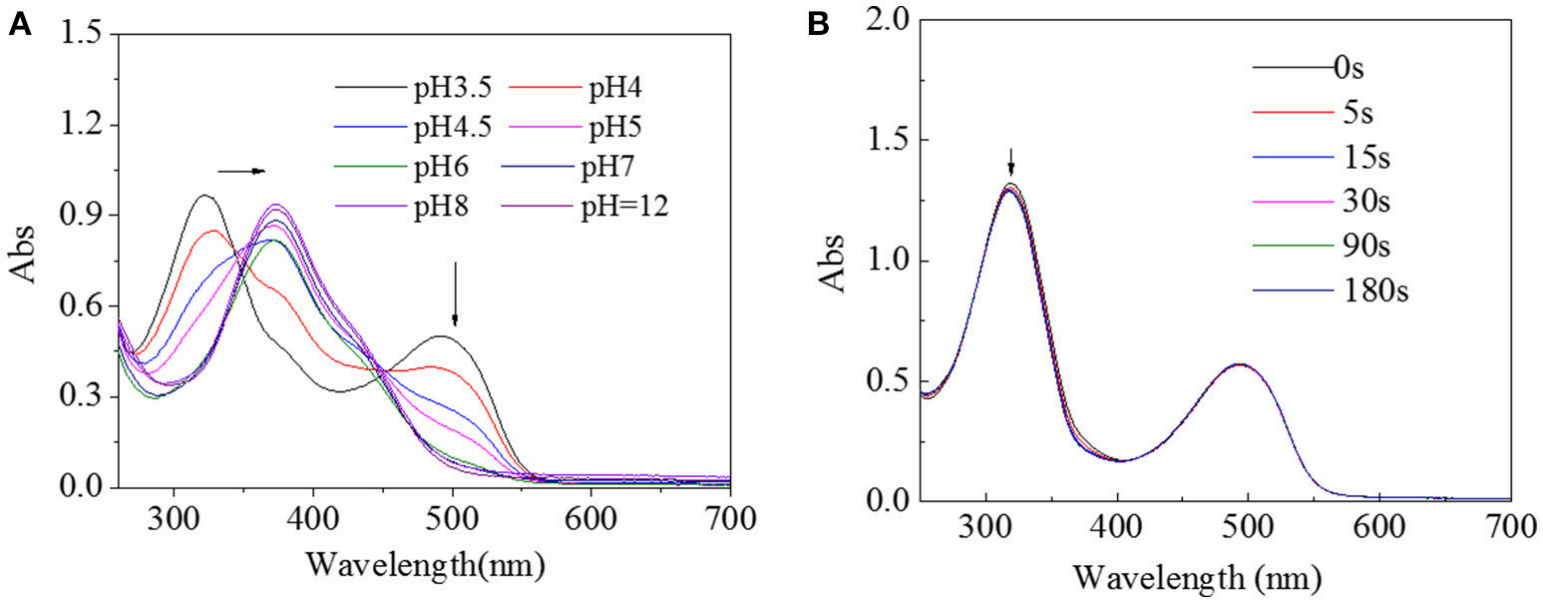
**(A)** UV spectra of HA-AZO water solution as a function of pH value. **(B)** UV spectra of HA-AZO water solution as a function of irradiation time in pH = 3.5 medium.

Since pH value is also an important factor in physiological environment, UV spectra of HA-AZO solution with different pH value was tracked as shown in Figure 3A. With decreasing pH value, the peak at 360 nm shifted to 320 nm and a new peak at 500 nm gradually increased when pH value was lower than 6. The blue shift from 360 to 320 nm was estimated to protonation effect of benzene ring for π-π^*^ transition and emerging peak at 500 nm was estimated to n-π^*^ transition in effects of protonation. In addition, in order to inspect the influence of irradiation on structure of HA-AZO, UV spectra of acid HA-AZO solution as a function of irradiation time was detected as shown in Figure 3B. It was found that the macromolecule has no responsive property in acid environment since UV irradiation had no influence on its UV spectra, which indicated that HA-AZO exhibited pH-dependent photoresponsive property. Theoretically, no visible trans-to-cis transition in acid environment might be the case, for two reasons. One is unstability of cis-form structure with very short existing time beyond characterization. The other one is protonation of AZO induces cis-form structure (from n-π^*^ transition) that coexists with trans-form structure in natural state, which indicates that trans-to-cis transition is induced in acid environment. However, no defined deduction has been verified by sufficient proof. Furthermore, since AZO molecule in water exhibited no trans-to-cis transition due to aggregation of molecules according to Figure S1, AZO molecule in water could not have pH-dependent photo responsive property.

**Figure 4:**
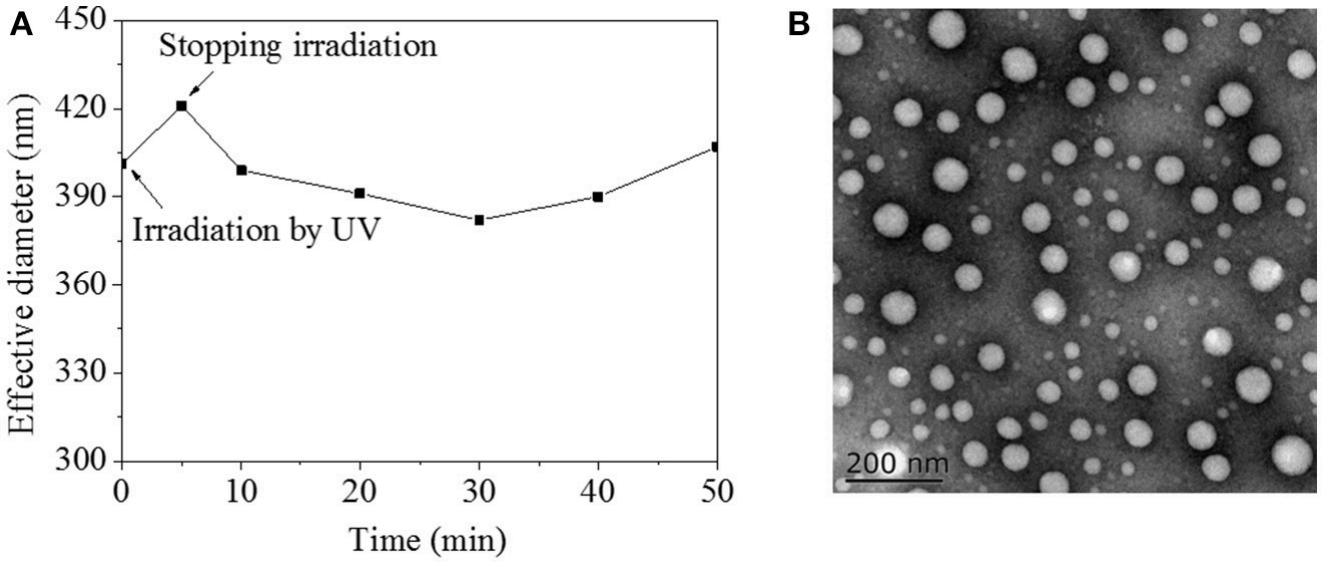
**(A)** Effective diameter of HA-AZO water solution a function of time by influence of UV irradiation; **(B)** TEM images of HA-AZO dropped by HA-AZO water solution and dried naturally.

On account of π-π stacking interaction between AZO domains, HA-AZO macromolecule might be in aggregation state, which was characterized by DLS and TEM as shown in Figure 4. Before irradiation, the effective diameter of HA-AZO solution was 405 nm (Figure 4A). The effective diameter increased after irradiation and then decreased gradually along with time until 30 min (Figure 4A). According to previous research, π-π stacking interaction between AZO molecules could be disaggregated when the trans-form transferred to cis-form, which made the interaction within aggregations weakened. The weaker interaction could lead aggregations to swell and dismiss at the end. However, since chain movement of macromolecule need time, recovery process of cis to trans came to procedure before aggregations dismissed completely. In recovery procedure, dismissed aggregations gradually reassembled again along with time. In further step, HA-AZO aggregations were observed by TEM as shown in Figure 4B. Spherical aggregations with diameter of 20–100 nm were homogeneously distributed in the TEM image. Because HA-AZO aggregations in water were in swollen state, diameter detected by DLS was much larger than that detected by TEM.

### Cytotoxicity of HA-AZO

**Figure 5:**
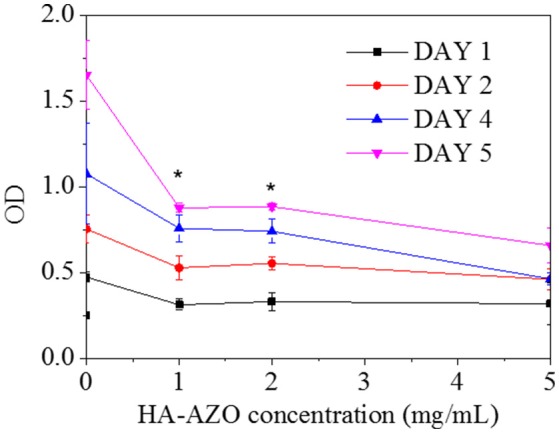
Optical density of HUVEC cells after cultured different time as a function of HA-AZO concentration. Cells was incubated with MTT.

Since biocompatibility of the macromolecule was an essential factor for its biomedical application, preliminarily cytotoxicity assessment was performed by *in vitro* culture of HUVEC cells. In this method, cells were cocultured with HA-AZO on TCPs, and simultaneously, cytoviability and cell apoptosis profiles were used to evaluate the biocompatibility of HA-AZO. As shown in Figure 5, the viability of cells decreased with HA-AZO concentration regardless of culture time. On the first day, OD of cells with HA-AZO showed no obvious decline, estimated 80% of that on TCPs even when HA-AZO concentration was as high as 5 mg/mL, which indicated that HA-AZO had no obvious acute cytotoxicity. However, cells with HA-AZO showed much lower OD than cells without HA-AZO after cells were cultured for some time. Moreover, OD of cells increased with cultured time, which indicated that cells proliferated along with time. However, it was inferred from OD statistic data that cells with HA-AZO showed slower proliferation rate than that of control (without HA-AZO), and the proliferation rate decreased along with HA-AZO concentration. These results indicated that proliferation of cells might be inhibited by HA-AZO. As a contrast, equivalent AZO exhibited great acute cytotoxicity and complete proliferation inhibition (Figure s4).

**Figure 6:**
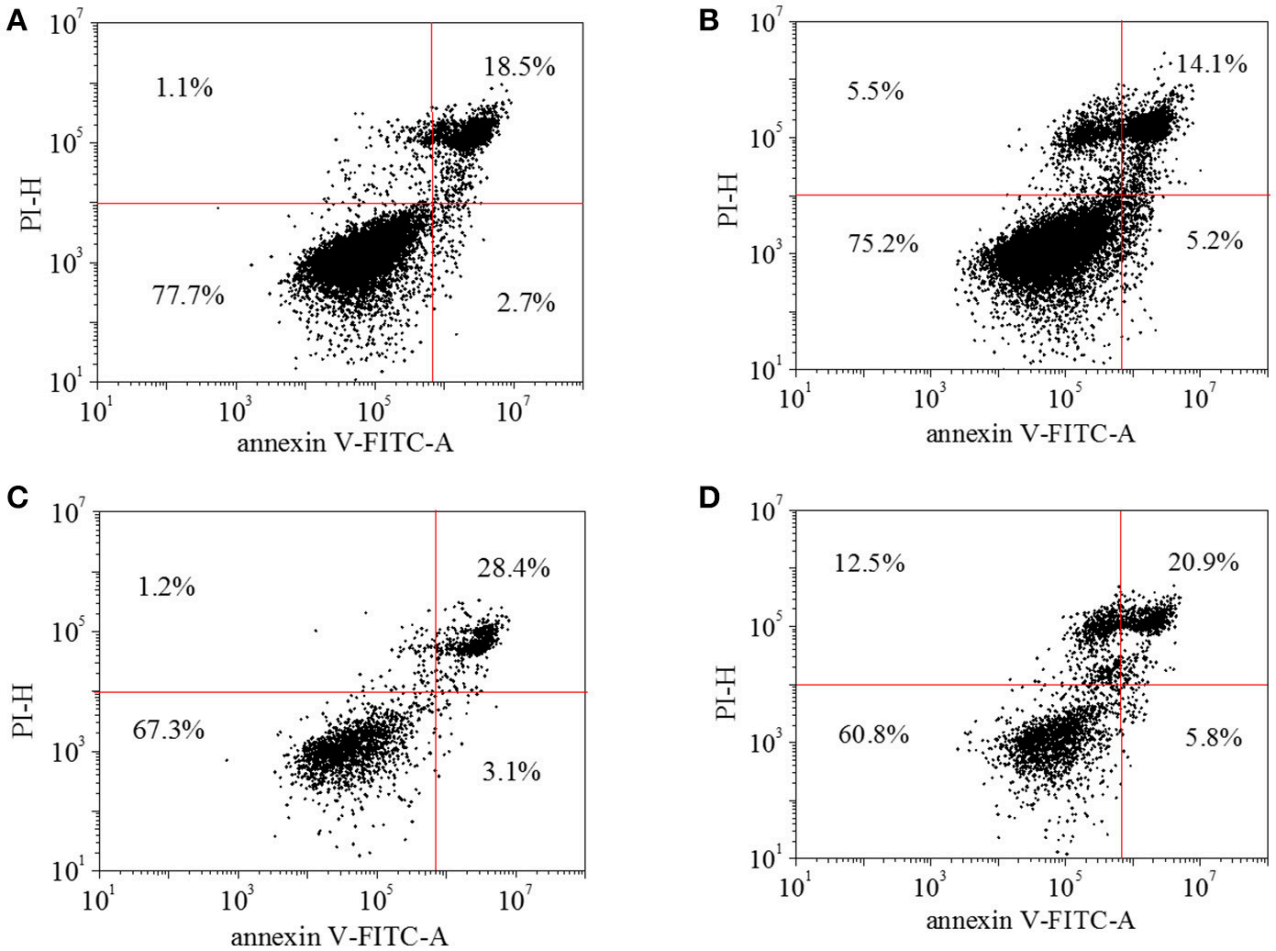
HUVEC cell apoptosis profile of with **(A)** 0 mg/mL HA-AZO, **(B)** 1 mg/mL HA-AZO, **(C)** 5 mg/mL HA-AZO, **(D)** 10 mg/mL HA-AZO. Cells was stained by FITC/PI.

In the cell apoptosis profile, live cells on TCPs without HA-AZO were approaching 78% and the dead cells were estimated to be 18% after cells were cultured for 3 days (Figure 6A). Further live cells on TCPs decreased with the increase of HA-AZO concentration (Figure 6B–D). Even when HA-AZO concentration was 5 mg/mL, live cells had been estimated 60%. The results were consistent with their viability as shown in Figure 5. As a contrast, equivalent AZO exhibited great cytotoxicity with less live cells and more dead cells in Figure s5.

## Conclusion

HA-AZO was successfully synthesized with DS of 2.2%. Synthesized HA-AZO had good water-solubility and a stable π-π^*^ transition absorbance peak trans-isomer. Upon UV for 5 min, transformation from trans-isomer to cis-isomer of HA-AZO could be realized according to UV spectra. Reversely, trans-isomer could be gradually recovered from cis-isomer in 1 h in dark. This characteristics revealed the reversible photo-responsive property of HA-AZO molecule with quick response and slow recovery. Moreover, antifatigue property of HA-AZO was confirmed by repeated illumination. The blue shift from 360 to 320 nm and emerging peak at 500 nm were found in low pH value solution. In acid solution, HA-AZO no longer showed any photo responsive property. HA-AZO was in small aggregation and its size was influenced by irradiation and varied with recovery time. Finally, *in vitro* evaluation showed little acute cytotoxicity, but the cell growth could be inhibited by HA-AZO. These properties indicated that HA-AZO was an optimal photo responsive macromolecule for biomedical application. The toxicity of AZO has been verified not only by our results but also by other researchers. Hence, their application in biomedical field is rarely reported. While the AZO grafting product HA-AZO exhibits little acute cytotoxicity from the result of both cell viability and cell apoptosis profile after 1 d, acute cytotoxicity is evaluated with 24 h before cell division. Theoretically, cell viability should multiply and increase with cultured days, since cells divide in every 24 h. Actually, cell growth viability showed little increase with cultured days. However, the increased rate is much lower than both that of TCPs control and theoretical calculation. These results showed that HA-AZO influenced cell proliferation and cell division, which indicated that the grafting product might have some genetic toxicity.

## Author contributions

JP designed the macromolecule and predicted its property. ZG synthesized the macromolecule. LZ and HW investigated the properties of the macromolecule. XH was responsible for the whole manuscript.

### Conflict of interest statement

The authors declare that the research was conducted in the absence of any commercial or financial relationships that could be construed as a potential conflict of interest.
