# Supplementary material for: Synthesis and Characterization of Photoresponsive Macromolecule for Biomedical Application
Source: Front Chem. 2018 Jul 2;6:217. doi: 10.3389/fchem.2018.00217 (PMC6036227; doi:10.3389/fchem.2018.00217)
Supplement: Supplementary file 1 [file Presentation_1.PDF]

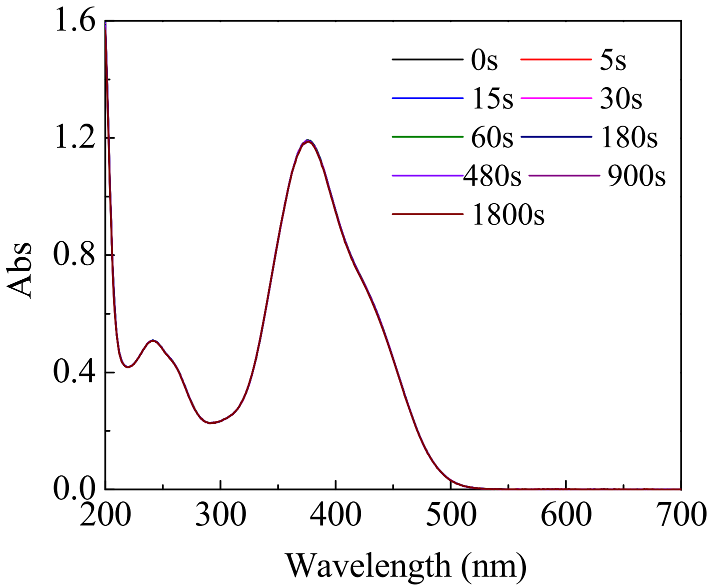

Fig. s1 UV spectra of AZO in water solution as a function of irradiation time

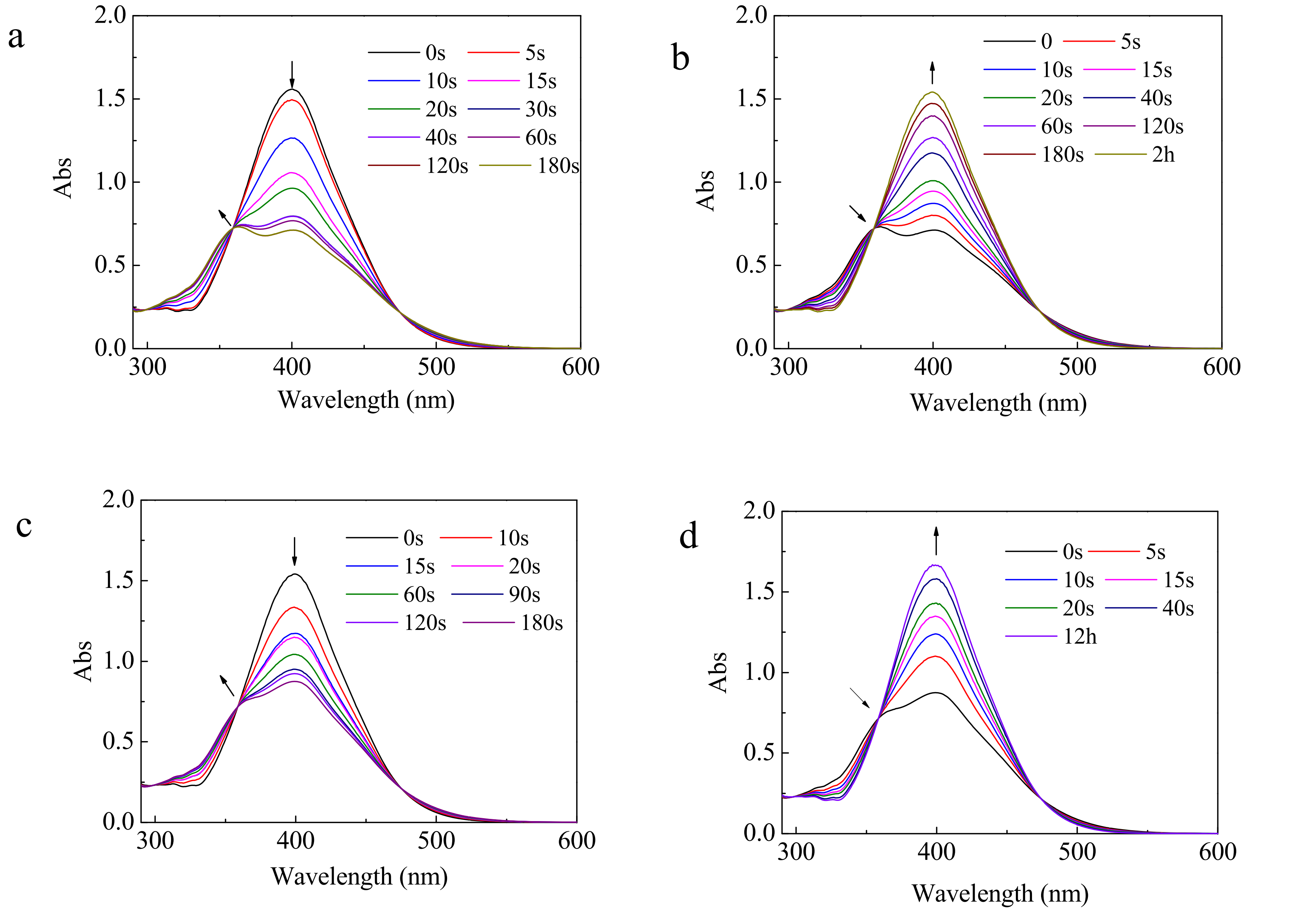

a
0s
5s
10s
15s
20s
30s
40s
60s
120s
180s
Abs
Wavelength (nm)
b
0
5s
10s
15s
20s
40s
60s
120s
180s
2h
Abs
Wavelength (nm)
c
0s
10s
15s
20s
60s
90s
120s
180s
Abs
Wavelength (nm)
d
0s
5s
10s
15s
20s
40s
12h
Abs
Wavelength (nm)

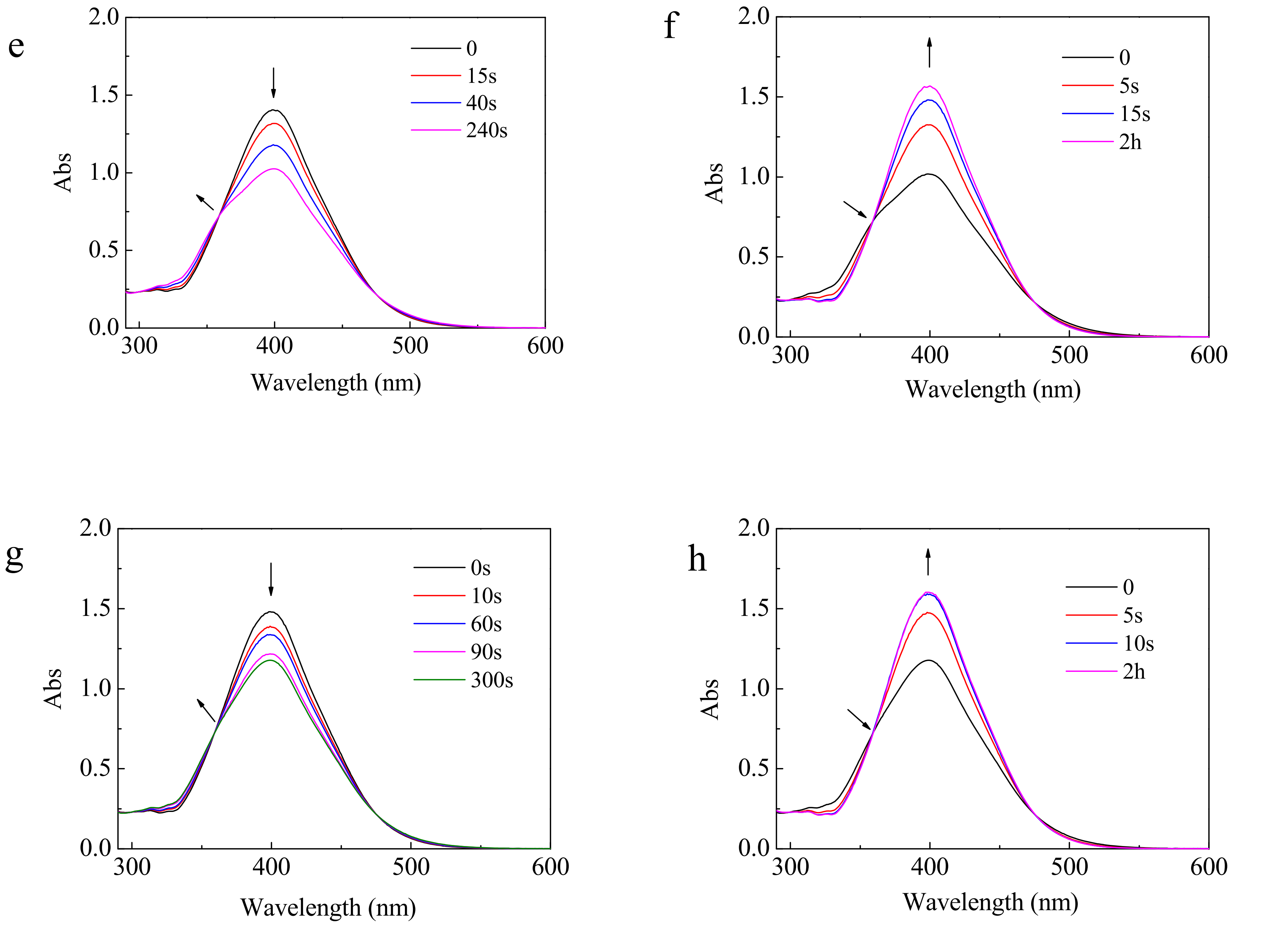
e
0
15s
40s
240s
Abs
Wavelength (nm)
f
0
5s
15s
2h
g
0s
10s
60s
90s
300s
h
0
5s
10s
2h

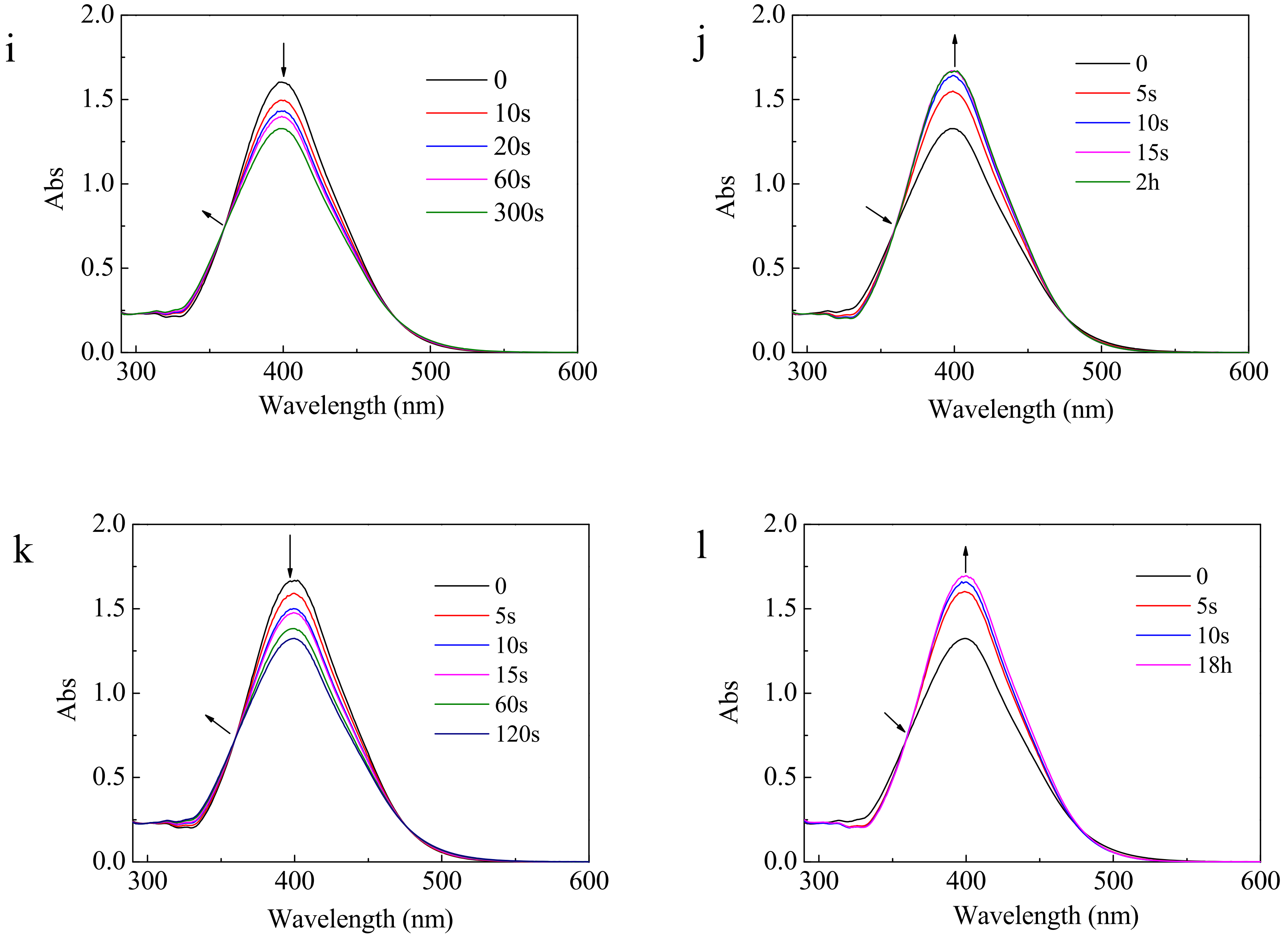

i
0
10s
20s
60s
300s
j
0
5s
10s
15s
2h
k
0
5s
10s
15s
60s
120s
l
0
5s
10s
18h
Abs
Wavelength (nm)

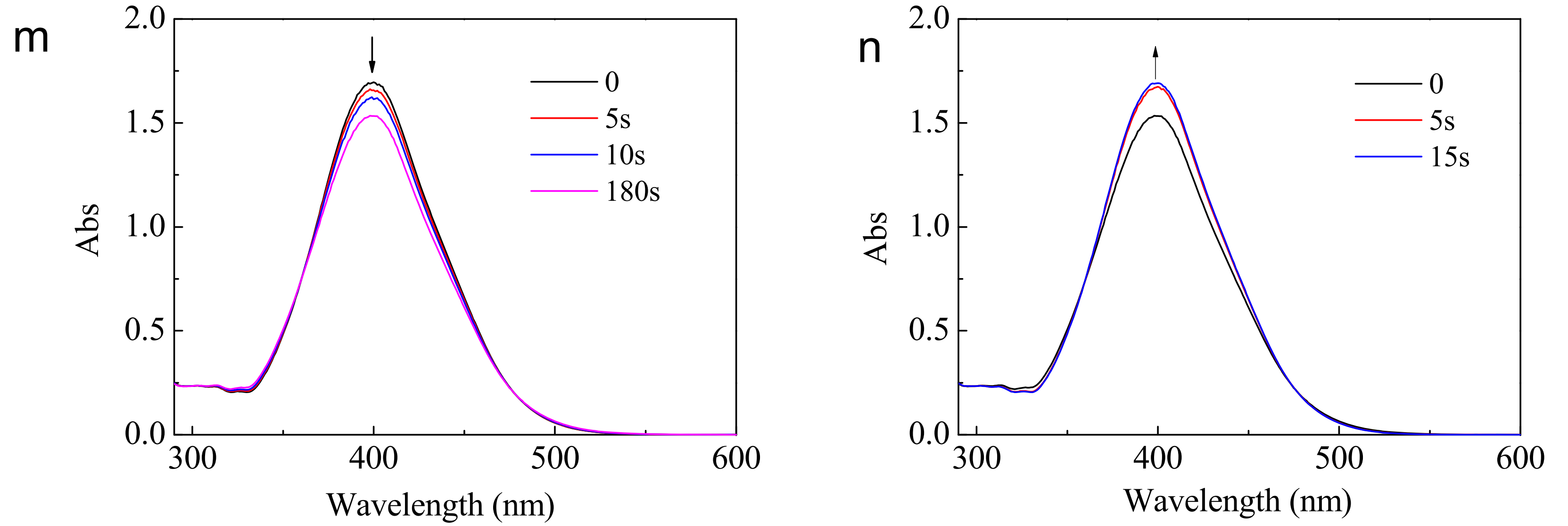

Fig. s2 UV spectra of AZO in DMF solution as a function of irradiation time (a,c,e,g,i,k,m) and recovery time (b,d,f,h,j,l,n) for different circle respectively. (a,b) first circle; (c,d)second circle; (e,f) third circle; (g,h) fourth circle; (i,j) fifth circle; (k,l) sixth circle; (m,n) seventh circle.

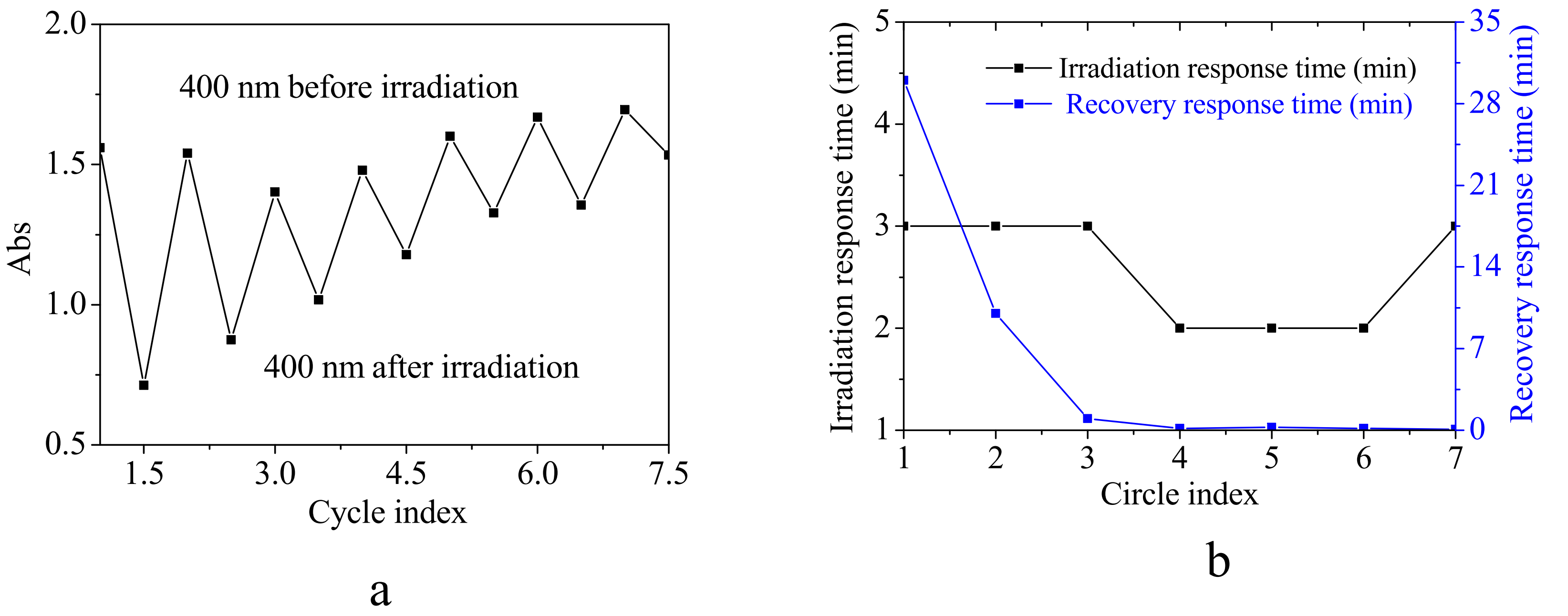

Fig. s3 (a) Absorbance at 400 nm of AZO in DMF solution as a function of circle index. (b) Irradiation response time and recovery response time in dark as a function of circle index

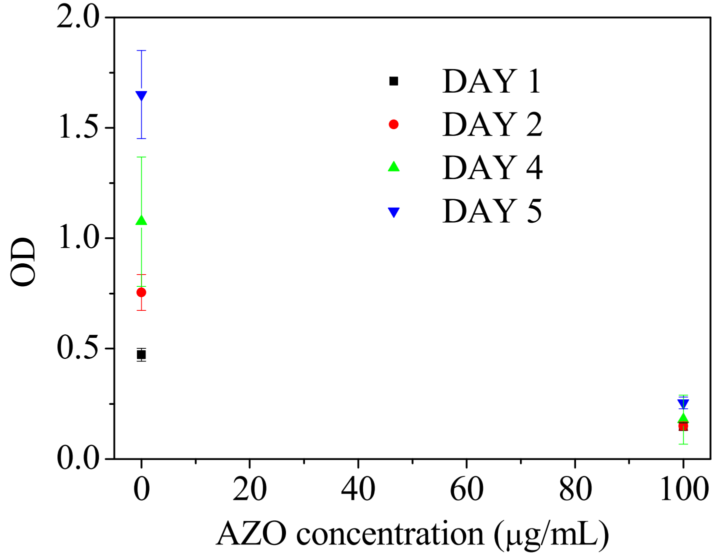

Fig.S4 Optical density of HUVEC cells after cultured different time as a function of AZO concentration. Cells was incubated with MTT.

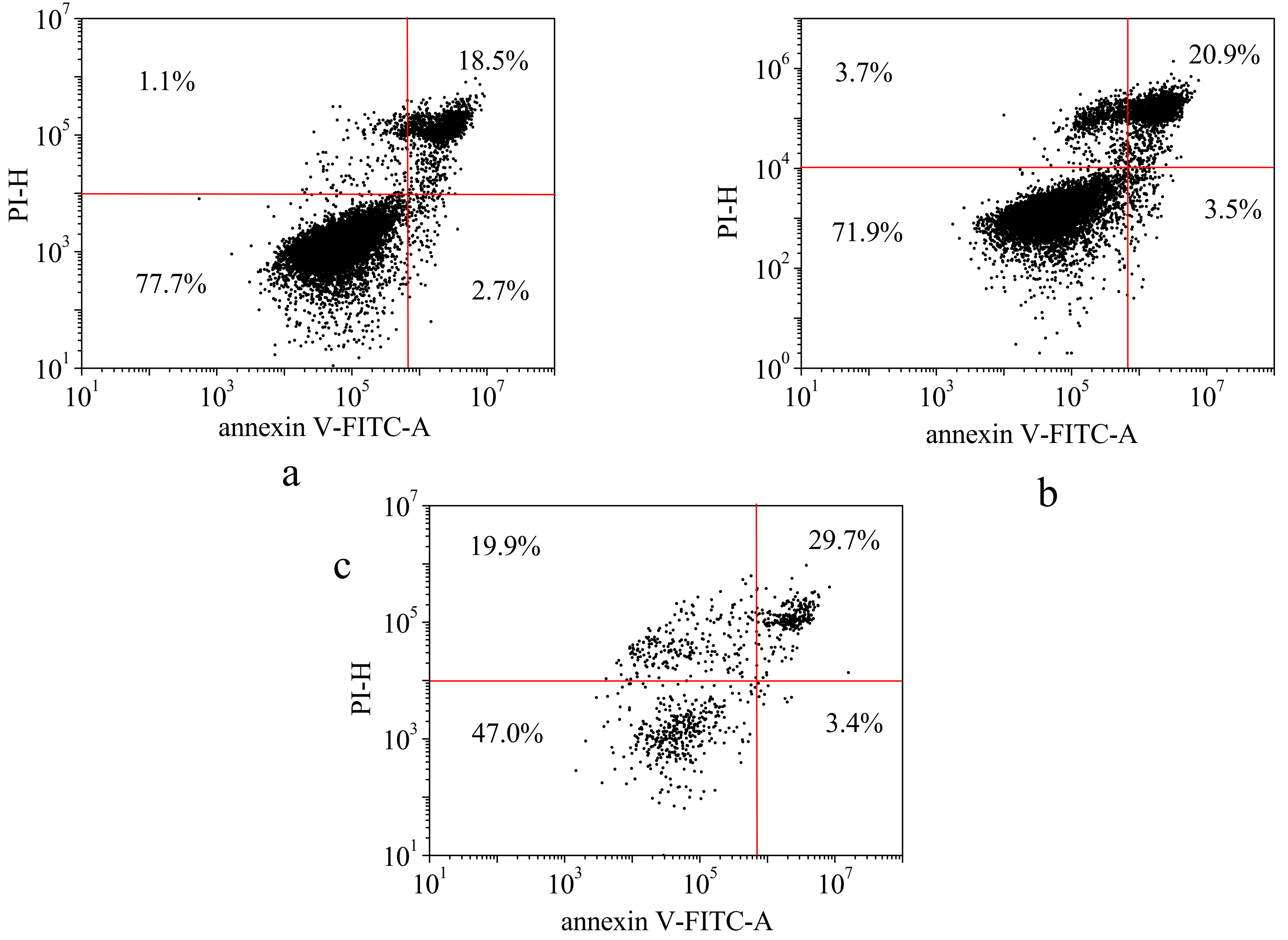

Fig.s5 cell apoptosis profile of with (a) 0mg/mL AZO, (b) 1mg/mL AZO, (c) 5mg/mL AZO. Cells was stained by FITC/PI
